# Lower than average HDL cholesterol efflux capacity in Lithuanian population

**DOI:** 10.1186/s12944-019-1124-2

**Published:** 2019-10-26

**Authors:** Sandra Kutkiene, Zaneta Petrulioniene, Dovile Karciauskaite, Aleksandras Laucevicius, Gabija Matuzevicienė, Justina Staigyte, Akvilė Saulyte Mikulskiene, Urte Gargalskaite, Egle Skiauteryte, Milda Kovaite

**Affiliations:** 1 Faculty of Medicine, Vilnius University, Santariškių 2, LT-08661 Vilnius, Lithuania; 2 Faculty of Medicine, Clinic of Cardiac and Vascular Diseases, Vilnius University, Vilnius, Lithuania; 3 Vilnius University Hospital Santaros Klinikos, Vilnius, Lithuania; 4 Faculty of Medicine, Department of Physiology, Biochemistry, Microbiology and Laboratory Medicine, Vilnius University, Vilnius, Lithuania

**Keywords:** HDL cholesterol efflux capacity, Severe dyslipidemia, HDL function, Residual risk

## Abstract

**Background:**

The aim of our study was to evaluate high-density lipoprotein cholesterol (HDL-C) efflux capacity in healthy controls and patients with severe dyslipidemia. Evaluation of HDL function may be beneficial for better understanding of cardiovascular diseases, as well as for taking actions to minimize residual cardiovascular risk.

**Methods:**

During 2016–2017 a total of 93 participants – 48 (51.6%) women and 45 (48.4%) men – were included in this cross-sectional study. Data of 45 (48.4%) participants with severe dyslipidemia (SD) and 48 (51.6%) controls without dyslipidemia was used for statistical analysis. Total lipid panel, concentration of lipoprotein (a) and apolipoproteins were measured, data about cardiovascular risk factors were collected and detailed evaluation of HDL-C quality was performed for all patients.

**Results:**

Increased HDL-C concentration was associated with higher ApoA1 (*r* = 0.866 in controls, *r* = 0.63 in SD group), ApoA2 (*r* = 0.41 in controls, *r* = 0.418 in SD group) and LDL-C concentrations (*r* = − 0.412 in SD group), lower ApoE (*r* = − 0.314 in SD group) and TG concentrations (*r* = − 0.38 in controls, *r* = − 0.608 in SD group), lower ApoB/ApoA1 ratio (*r* = − 0.567 in control group), below average HDL-C efflux capacity (*r* = − 0.335 in SD group), lower BMI (*r* = − 0.327 in controls, *r* = − 0.531 in SD group) and abdominal circumference (*r* = − 0.309 in women with SD). Below-average HDL-C efflux capacity was found in 67.7% (*N* = 63) of participants. It was more often found among patients with normal weight or BMI 30–31 kg/m2. HDL-C efflux capacity was inversely associated with HDL-C concentration (*r* = − 0.228).

**Conclusion:**

Abnormal HDL function may be associated with residual cardiovascular risk in Lithuanian population.

## Background

Cardiovascular disease accounts for 65% of deaths among women and 48% among men [1]; according to EUROSTAT, Lithuania recorded the highest rate of death due to ischemic heart disease in Europe [2]. According to the Lithuanian High Cardiovascular Risk (LitHiR) primary prevention program, the prevalence of any type of dyslipidemia in Lithuania is high – 89.7% [3], although only 13.7% of middle-aged Lithuanians have low HDL-C [4]. HDL is responsible for many actions related to maintaining arterial wall homeostasis, such as reverse cholesterol transport, anti-inflammatory, antioxidative, anti-apoptotic, anti-infectious, anti-thrombotic and vasodilatory effects [5, 6]. HDL-C cardio-protective role has been supported by large population studies [7, 8]. It was estimated that by increasing HDL-C concentration by 0.026 mmol/l (1 mg/dL), cardiovascular disease risk decreases by 2% in men and 3% in women [9]. On the other hand, some novel studies show attenuated relationship between low HDL-C and coronary heart disease (CHD) risk [10]. The relationship between HDL-C levels and CHD incidence does not seem to be causal, both according to Mendelian randomization studies [11], and based on the fact that drugs targeted to increase HDL-C concentration failed to show benefit in randomized controlled trials [12, 13], [14]. In addition, Danish population studies revealed that both low and high HDL-C concentration were associated with higher all cause and cardiovascular mortality [15]. Individuals with certain genetic variants and very high HDL-C concentration may as well develop atherosclerosis, which could be explained by abnormal HDL function [16]. Better understanding of HDL function would be of great benefit, as impaired HDL function may be associated with residual cardiovascular risk. There are multiple laboratory assays for evaluation of various cardio-protective effects of HDL. HDL-C efflux capacity assay evaluates how HDL removes cholesterol from cells, including macrophages, through ABCA1, and represents a major athero-protective role of HDL particles, as shown in vivo [17] and in real life data, since HDL-C efflux capacity is significantly and inversely associated with incident coronary heart disease events, independent of several established cardiovascular risk factors [18]. The aim of our study was to evaluate HDL-C efflux capacity in healthy participants and patients with severe dyslipidemia.

## Methods

During 2016–2017 a total of 93 participants (48 (51.6%) women and 45 (48.4%) men) from 18 up to 60 years of age with severe dyslipidemia (SD) or no lipid abnormality (control group) without possible causes of secondary dyslipidemia and evident cardiovascular disease were included in this cross-sectional study. The study was approved by the Local Research Ethics Committee, and written informed consent was obtained from each participant before including them into the study. Data of 45 (48.4%) participants with severe dyslipidemia (SD) and 48 (51.6%) controls without dyslipidemia, overt cardiovascular disease and disorders that may cause secondary dyslipidemia, were collected and detailed evaluation of the HDL-C quality was performed.

SD was defined as serum total cholesterol ≥7.5 mmol/L (290 mg/dL), or low-density-lipoprotein cholesterol (LDL-C) ≥6 mmol/L (232 mg/dL). Patients with overt cardiovascular disease and possible secondary dyslipidemia were not included into the study (uncontrolled hypothyroidism, diabetes mellitus, nephrotic syndrome, renal insufficiency, cholestasis, viral hepatitis, liver cirrhosis, alcoholism, anorexia, pregnancy, terminal stage cancer and any terminal). Controlled thyroid dysfunction and diabetes mellitus diagnosed later than dyslipidemia were not considered as exclusion criteria. Only subjects without dyslipidemia, evident cardiovascular disease (myocardial infarction, unstable angina, stable angina with positive cardiac stress test, coronary artery pathology identified during cardiac catheterization or coronary computed tomography angiography, coronary artery bypass surgery, percutaneous coronary intervention), cerebrovascular disorder (previous acute ischemic or hemorrhagic stroke, diagnosed stenosis of carotid arteries), peripheral artery disease (acute ischemic syndromes, chronic limb ischemia, aortic aneurysm), disorders that may impact concentrations of blood lipids were included in control group of the study. If patient had SD and treatment with statins or other drugs was provided but lipid profile was still abnormal (fulfilling criteria of severe hypercholesterolemia), patients were included into the study. We did not differentiate patients into SD without treatment group or SH with insufficient treatment.

Total cholesterol, LDL-C, HDL-C, triglycerides (TG), apolipoprotein A1 (ApoA1), apolipoprotein A2 (ApoA2), apolipoprotein B (ApoB), apolipoprotein E (ApoE) and lipoprotein (a) (Lp(a)) concentrations, as well as ApoB/ApoA1 ratio were measured. All the tests were carried out in the morning and participants were advised not to eat for at least 12 h. Data about cardiovascular risk factors, such as arterial hypertension, smoking, alcohol consumption, familial history of CHD and diabetes mellitus in first degree relatives, insufficient physical activity and body mass index (BMI) were collected.

While assessing the BMI, the following groups were identified: ideal – 22, normal – 20–25 for men and 18.5–24 for women, overweight – 25–29.9, obese – 30–40 and severely obese – > 40.

Arterial hypertension was considered as systolic blood pressure ≥ 140 mmHg and/or diastolic blood pressure ≥ 90 mmHg, or the diagnosis of hypertension was documented in a medical record.

Insufficient physical activity was described as exercises less than 45 min 3 times a week.

HDL-C efflux capacity was evaluated by using *Cholesterol efflux fluorometric assay kit (BioVision, Inc., CA, USA*) according to manufacturer’s protocol. As HDL cholesterol efflux capacity assay does not have standardized reference range, HDL cholesterol efflux was calculated and categorized into tertiles – below average, average and above average.

### Statististics

Categorical variables were described through frequencies (%) and continuous variables were expressed by means and standard deviations (SD). Continuous variables were compared using the Kruskal–Wallis univariate analysis of variance (ANOVA). Categorical variables were compared using the *Chi-square* test or *Fisher* exact test. In order to assess the linear association between characteristics, correlation analysis was performed and Spearman’s correlation coefficient (r) was applied. Correlation was defined as weak (r < 0.3), moderate (0.3 ≤ r ≤ 0.7) or strong (r > 0.7). A *p*-value of < 0.05 was considered significant. Statistical analyses were performed using SPSS 23.0 (SPSS Inc., Chicago, Illinois, US).

## Results

### General characteristics

The baseline characteristics of the participants are provided in Table 1. There was a significant age difference in the whole study population between participants in SD and control groups (50.53 ± 1.11 years vs. 47.04 ± 0.76 years, *p* = 0.001). More patients with severe dyslipidemia had arterial hypertension (44.4% vs. 20.8%, *p* = 0.015), and family history of CHD (64.4% vs. 12.5%, *p* < 0.001), compared to control group (Table 1).

**Table 1:** The baseline characteristics and trends of cardiovascular risk factors of the study population (*n* = 93)

Characteristics	All patients		Severe dyslipidemia group		Control group		*p*
	*n* = 93		*n* = 45		*n* = 48		
	Mean	SD	Mean	SD	Mean	SD	
Age (years)	48.73	6.60	50.53	1.11	47.04	0.76	0.001
TC (mmol/l)	6.16	2.32	7.93	0.32	4.51	0.08	< 0.001
LDL-C (mmol/l)	4.05	2.06	5.42	0.33	2.77	0.07	< 0.001
HDL-C (mmol/l)	1.28	0.32	1.26	0.05	1.30	0.04	0.975
TG (mmol/l)	1.77	1.82	2.63	0.34	0.95	0.06	< 0.001
ApoA1 (mmol/l)	1.66	0.25	1.68	0.04	1.64	0.03	0.321
ApoB (mmol/l)	1.10	0.49	1.47	0.07	0.75	0.02	< 0.001
ApoA2 (mmol/l)	0.34	0.05	0.35	0.01	0.33	0.01	0.051
ApoB/ApoA1	0.66	0.29	0.88	0.04	0.46	0.01	< 0.001
ApoE (mg/l)	56.43	29.08	71.69	4.93	42.13	2.03	< 0.001
Lp(a) (g/l)	0.19	0.28	0.25	0.05	0.13	0.03	0.003
*Frequencies*	n	%	n	%	n	%	*p*
Men (%)	45	48.4	21	46.7	24	50	0.748
AH (%)	30	32.3	20	44.4	10	20.8	0.015
Abdominal obesity (%)	44	47.3	22	48.9	22	45.8	0.768
Smoking (%)	30	32.3	12	26.7	18	37.5	0.264
CHD history (%)	35	37.6	29	64.4	6	12.5	< 0.001

*Abbreviations: SD* standard deviation, *TC* total cholesterol, *LDL-C* low-density lipoprotein cholesterol, *HDL-C* high-density lipoprotein cholesterol, *TG* triglycerides, *Apo* apolipoprotein, *Lp(a)* lipoprotein(a), *AH* arterial hypertension, *CHD* coronary heart disease

### HDL-C concentration

No significant difference in HDL-C concentration was found between control group and SD group (1.26 ± 0.32 mmol/l vs. 1.30 ± 0.04 mmol/l, *p* = 0.975). Decreased HDL-C (< 1.2 mmol/l (46 mg/dL) in women and < 1 mmol/l (39 mg/dL) in men [19]) was found more frequently in participants with SD than in healthy controls (31% (*N* = 14) vs. 14.6% (*N* = 7), *p* = 0.004). We found 70.2% (*n* = 33) of decreased HDL-C concentrations in the SD group. Gender-specific analysis showed that more men with SD had decreased HDL-C (36.4%, *n* = 8) compared to the control group (13.6%, *n* = 3), *p* = 0.013, while no significant difference in HDL-C levels between women with SD (26%, *n* = 6) and healthy women (12.5%, *n* = 3) was found (*p* = 0.118).

A weak but significant correlation between age and HDL-C concentration was found (*r* = 0.180, *p* = 0.008). It was estimated that chances of severe dyslipidemia are approximately two times higher (OR 2.433, CI:1.366–4.334) when HDL-C < 1.19 mmol/l (46 mg/dL) (sensitivity – 46.4%, specificity – 73.8%, area under the curve – 2.6%).

For further analysis and in order to define increased, normal and decreased HDL-C concentration we calculated 33th percentiles, which divided all measurements into three equal parts. Decreased HDL-C concentration was defined as < 1.08 mmol/l (42 mg/dL), normal HDL-C concentration 1.08–1.4 mmo/l (42–54 mg/dL) and increased HDL-C concentration is > 1.40 mmol/l (> 54 mg/dL).

**Fig. 1:**
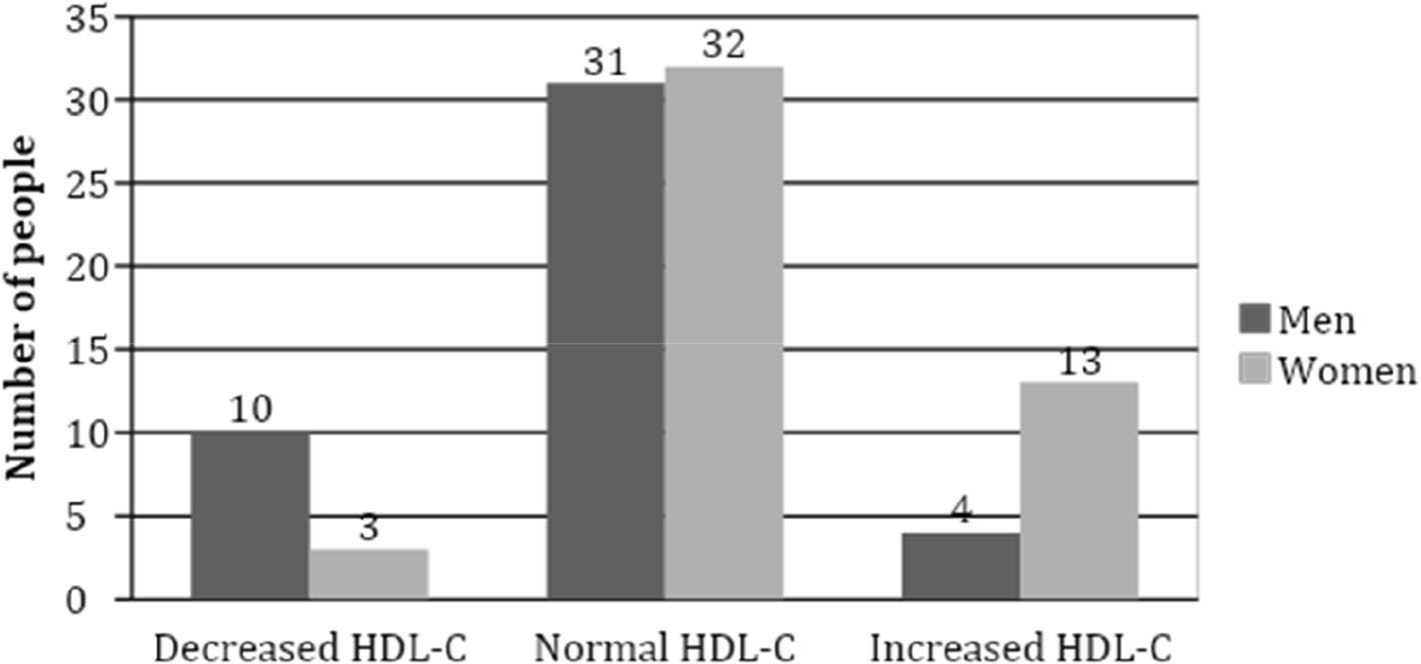
Association between HDL-C concentration and gender in the study population, *p* = 0,015 (*n* = 93). Decreased HDL-C concentration was defined as < 1.08 mmol/l, normal HDL-C concentration 1.08–1.4 and increased HDL-C concentration > 1.40. Decreased HDL-C was more frequently found among men

The majority of the participants (67.7%, *N* = 64) had normal value of HDL-C, irrespective of their SD status. Decreased HDL-C concentration was found in 14.0% (*N* = 13) of the participants. Increased HDL-C concentration was found in 18.3% (*N* = 17) of the subjects, more often in women than men (27% *N* = 13 vs. 8.9% *N* = 4, *p* = 0.015) (Fig. 1). There was no significant difference in HDL-C concentration in different age groups.

### Correlation between HDL-C concentration and other cardiovascular risk factors

**Fig. 2:**
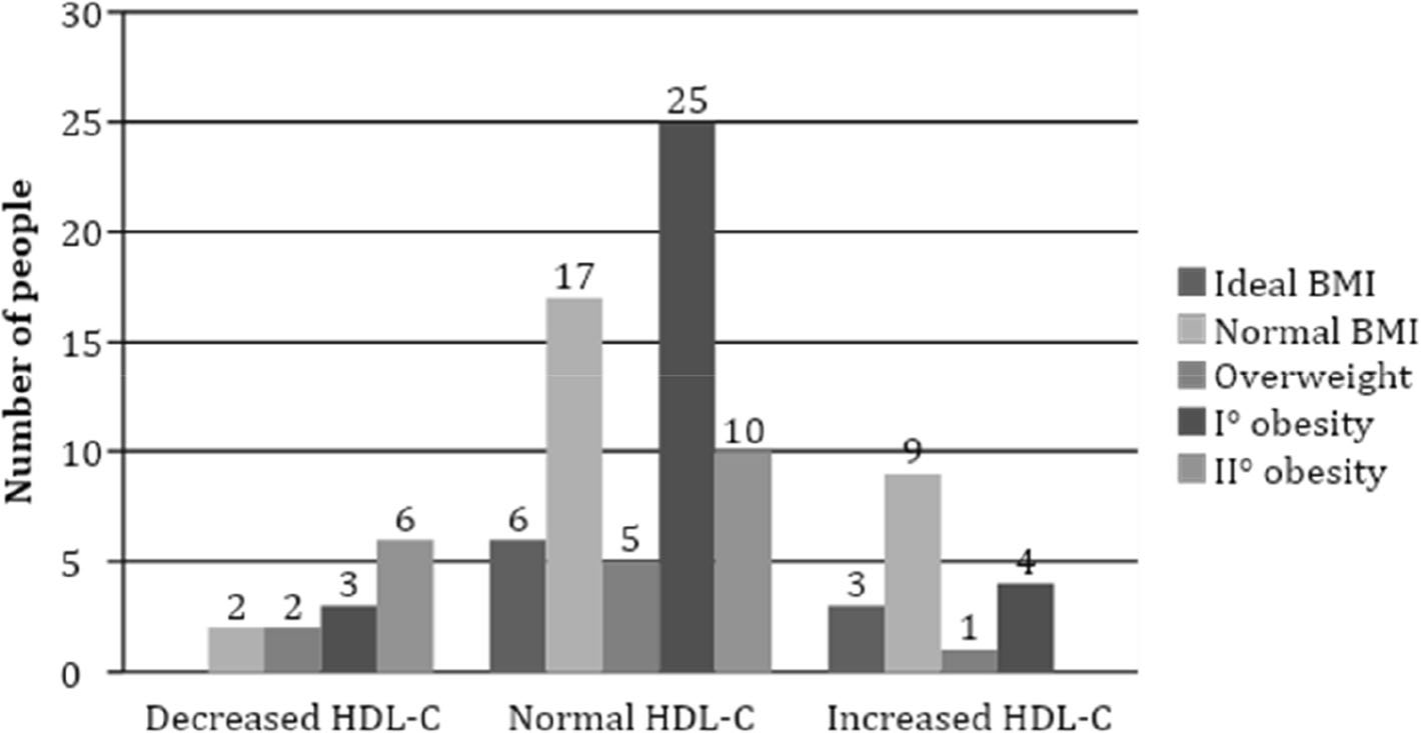
Association between HDL-C concentration and BMI in the study population, *p* = 0.02 (*n* = 93). Ideal BMI – 22, normal – 20–25 for men and 18.5–24 for women, overweight – 25–29.9, I° obesity – 30–40 and II° obesity – > 40. Normal HDL-C concentration was more often found in participants with I° obesity (*p* = 0.02)

**Fig. 3:**
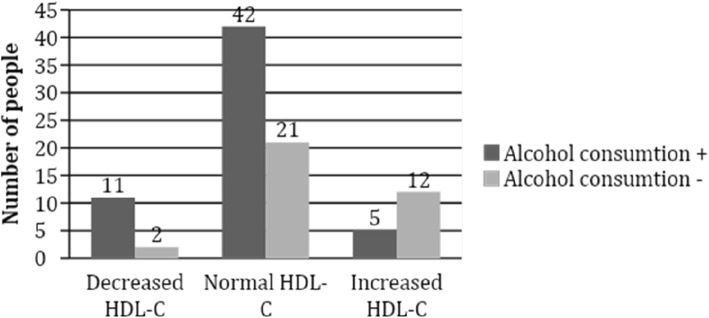
Association between HDL-C concentration and alcohol consumption in the study population, *p* = 0.004 (*n* = 93). Normal HDL-C concentration was more often found in participants who consumed alcohol, in comparison with those who did not consume alcoholic beverages (*p* = 0.004)

Normal HDL-C concentration was more often found in participants with class I obesity (*p* = 0.02) (Fig. 2) and those who consumed alcohol, in comparison with those who did not consume alcoholic beverages (*p* = 0.004) (Fig. 3). No association was found between HDL-C concentration and cardiovascular risk factors, such as abdominal obesity, arterial hypertension, smoking and familial history of coronary heart disease. Although physically active subjects tended to have normal levels of HDL-C more frequently than participants with insufficient physical activity, no significant differences were found.

Strong statistically significant positive correlation between HDL-C and ApoA1 concentration was found in participants with SD (*r* = 0.63) and control group (*r* = 0.866) (Table 2). The correlation was significant in all age groups. Higher HDL-C was also associated with higher LDL-C concentration in participants with SD (*r* = 0.412) and higher ApoA2 concentration in participants with SD (*r* = 0.418) and control group (*r* = 0.41).

**Table 2:** Spearman’s correlation coefficients between HDL-C concentration and other characteristics in groups with and without severe dyslipidemia

Characteristics	HDL-C (mmol/l)			
	SD–	SD+	SD+	
			Men	Women
HDL-C efflux capacity (%)	− 0.146	− 0.335*	− 0.123	− 0.198
Age (years)	0.130	0.061	0.059	−0.030
TC (mmol/l)	0.342*	0.277	−0.008	0.327*
TG (mmol/l)	−0.380*	− 0.608*	− 0.582*	− 0.217
LDL-C (mmol/l)	− 0.057	0.412*	0.083	0.232
Apo A1 (mmol/l)	0.866*	0.630*	0.713*	0.755*
Apo B (mmol/l)	−0.097	0.275	−0.065	0.126
Apo A2 (mmol/l)	0.410*	0.418*	0.317*	0.455*
Apo E (mmol/l)	0.140	−0.314*	−0.160	0.050
Apo B/Apo A1	−0.567*	−0.015	− 0.325*	−0.104
Lp(a) (g/l)	−0.263	0.232	−0.141	−0.015
BMI (kg/m2)	−0.327*	−0.531*	− 0.461*	−0.441*
WC (cm)	0.183	−0.583*	−0.189	− 0.309*

*Abbreviations: HDL-C* high-density lipoprotein cholesterol, *TC* total cholesterol, *TG* triglycerides, *LDL-C* low-density lipoprotein cholesterol, *Apo* apolipoprotein, *Lp(a)* lipoprotein(a), *BMI* body mass index, *WC* waist circumference

* Statistically significant as *p* < 0.05

HDL-C concentration was inversely associated with ApoE (*r* = − 0.314) and HDL-C efflux capacity (*r* = − 0.335) in participants with SD. In addition, there was inverse association between HDL-C and TG concentration in men with SD (*r* = − 0.582) and control group (*r* = − 0.38) in all age groups, and APOB/APOA1 ratio in control group (*r* = − 0.567); this association decreased with age (Table 3). Lower HDL-C was also associated with higher BMI in participants with SD (*r* = − 0.531) and control group (*r* = − 0.327) and higher waist circumference in women with SD (*r* = − 0.309). Age did not impact HDL-C concentration in this analysis.

**Table 3:** Spearman’s correlation coefficients between HDL-C concentration and other characteristics in different age groups

Characteristics	HDL-C (mmol/l)		
	<45y	45-54y	≥55y
HDL-C efflux capacity (%)	−0.217	− 0.267	0.006
Age (years)	− 0.330	0.282	−0.365
TC (mmol/l)	0.110	0.226	−0.177
TG (mmol/l)	−0.588*	−0.326*	− 0.775*
LDL-C (mmol/l)	0.032	0.168	−0.083
Apo A1 (mmol/l)	0.864*	0.669*	0.803*
Apo B (mmol/l)	0.127	0.065	−0.349
Apo A2 (mmol/l)	−0.014	0.600*	0.511
Apo E (mg/l)	−0.108	0.040	−0.481
Apo B/Apo A1	−0.374*	−0.143	− 0.535
Lp(a) (g/l)	−0.295	0.138	0.194
BMI (kg/m2)	−0.561*	−0.400*	− 0.398
WC (cm)	−0.272	− 0.273	−0.209

*Abbreviations: HDL-C* high-density lipoprotein cholesterol, *TC* total cholesterol, *TG* triglycerides, *LDL-C* low-density lipoprotein cholesterol, *Apo* apolipoprotein, *Lp(a)* lipoprotein(a), *BMI* body mass index, *WC* waist circumference

* Statistically significant as *p* < 0.05;

### HDL cholesterol efflux capacity

**Fig. 4:**
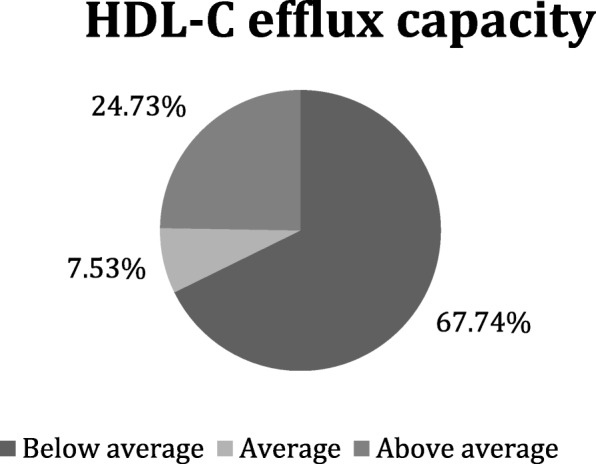
Evaluation of HDL-C efflux capacity (%) in the study population (*n* = 93). Majority of participants (67.74%) had below average HDL-C efflux capacity

The average HDL-C efflux capacity in the study population was 47.5%. Below-average HDL-C efflux capacity was found in 67.7% (*N* = 63) of the participants (Fig. 4). Significant differences in HDL-C efflux capacity between men and women were not detected. Women with SD more often had below-average HDL-C efflux capacity (75% (*N* = 36) vs 60% (*N* = 27), *p* = 0.238), however the difference was not statistically significant.

Statistically significant negative correlation between HDL-C efflux capacity and HDL-C concentration was found (*r* = − 0.228). Correlation was not significant when evaluated separately in men and women cohorts. No significant correlation between SD and HDL-C efflux capacity was found.

Below-average HDL-C efflux capacity was more frequently found in participants with normal BMI and class I obesity compared to overweight participants and those with class II obesity (*p* ≤ 0.05). It was observed that men with SD and normal weight had impaired HDL-C efflux capacity more frequently. No relation was found between HDL-C efflux capacity and participants’ gender or cardiovascular risk factors, such as abdominal obesity, arterial hypertension, familial history of coronary heart disease and alcohol consumption. Although below-average HDL-C efflux capacity was more common among physically active subjects compared to patients with insufficient physical activity, this difference was not statistically significant.

## Discussion

According to LitHiR Primary Prevention Program, the prevalence of any type of dyslipidemia in middle-aged population (50–65 years old women and 40–55 years old men) is high – 89.7% [3], while the prevalence of low HDL-C is unexpectedly low – 13.7% [4]. It was estimated that average HDL-C concentration is 1.54 ± 0.46 mmol/l (59.6 ± 17.8 mg/dL) [3]. While in Poland NATPOL 2011 representative 40–59 years sample, prevalence of low-HDL was 35.2% in men and 22.2% in women, average HDL was 1.22 mmol/l (47.3 mg/dL) in men and 1.41 mmol/l (54.7 mg/dL) in women [20]. Interestingly participants of LitHiR with isolated low HDL-C had more favorable risk profile than participants with other lipid abnormalities, such as isolated triglyceridemia and atherogenic dyslipidemia [21]. In addition, recent studies suggest that low HDL-C may paradoxically be associated with reduced risk of incident CHD in black participants population [22], which led us to investigate the quality of HDL-C.

According to two prospective Danish cohort studies the HDL cholesterol concentration associated with the lowest risk of all-cause mortality is 1.9 mmol/L (73 mg/dL) for men, and 2.4 mmol/L (93 mg/dL) for women [15]. Although some researchers define an HDL-C of 1.55 mmol/l (60 mg/dL) or greater as high HDL-C and an HDL-C of 2.59 mmol/l (100 mg/dL) or greater as very high HDL-C [23], there is no consensus on what HDL-C is “too high”. In our study due to small sample size for statistical analysis sample was divided into 3 groups according to 33 percentiles and increased HDL-C was defined as HDL-C greater than 1.4 mmol/l (54 mg/dL).

According to literature, HDL-C concentration is inversely associated with weight, abdominal circumference, TG concentration, number of small dense LDL particles, systemic inflammatory response and smoking [24]. Both extremely low and high HDL-C levels are associated with greater risk of all-cause, CHD and stroke mortalities and higher level of inflammatory factors [25], while the impact on risk of cancer is ambiguous [25], [22]. In our study low HDL-C was associated with higher BMI, abdominal circumference and TG. On the other hand, physical fitness is associated with higher HDL-C [26, 27]. This emphasizes the importance of lifestyle intervention in treating low HDL-C. It has been reported that 5–10% weight loss can increase HDL-C approximately 8–10% [28]. In our study physically active subjects tended to have normal levels of HDL-C more frequently than participants with insufficient physical activity, however this difference was not statistically significant.

Walter reported that plasma HDL-C levels change with age. In males HDL-C levels decrease during adolescence and early adulthood, but in elder age they are stable or even slightly increased [29]. In contrast, women’s HDL-C levels remain stable throughout their lifetime, however, menopause often causes a slight decrease in HDL-C concentration [30]. In our study a weak but significant correlation between age and HDL-C concentration was found. However, the differences of HDL-C levels in different age groups of both men and women were statistically insignificant when calculated with ANOVA.

ApoA1 accounts for approximately 70% of HDL total protein mass, and ApoA2 for 15–20%. Other proteins, such as ApoC, ApoD, ApoM and ApoA-IV, cholesteryl ester transfer protein (CEPT), and lipolytic enzymes, such as paraoxonase-1, glutathione peroxidase and platelet-activating factor acetylhydrolase (PAF-AH), as well as cholesterol acyl transferase form the remaining 15% [31, 32]. ApoA1 plays a major role in reverse cholesterol transport and is a major anti-atherogenic and antioxidant factor of HDL-C [31]. ApoA2 is also associated with reverse cholesterol transport and antioxidant potential [33]. ApoE is responsible for receptor-mediated uptake of lipoprotein, antioxidant, anti-inflammatory and endothelial activity. It induces release of endothelial nitric oxide [31]. In our study HDL-C concentration was positively associated with ApoA1, ApoA2 and inversely associated with ApoE concentration and APOB/APOA1 ratio.

In this study we evaluated HDL-C efflux capacity in healthy participants and patients with severe dyslipidemia. It was found that the majority of participants (67.7%) had below-average HDL-C efflux capacity. According to literature, there is a positive correlation between HDL-C efflux capacity and alcohol intake, and inverse correlation with type 2 diabetes and measures of obesity [18]. In our study HDL-C efflux capacity was not associated with traditional cardiovascular risk factors, except obesity (below-average HDL-C efflux capacity was more frequently found in participants with normal BMI and I° obesity compared to overweight participants and those with II° obesity). These are similar findings to Rohatgi et al. study, in which HDL cholesterol efflux capacity was minimally associated with cardiovascular risk factors [34]. However, our study did not reveal such tendencies. HDL-C efflux capacity was inversely associated with HDL-C concentration (*r* = − 0.228). Similar results were found in case control study of individuals with extremely high HDL-C and CAD [35].

It is still widespread myth in Lithuania that high HDL-C compensates effects of other lipid abnormalities. However even though average HDL-C concentration in Lithuanian middle age population is high, majority of participants in our study had below average HDL-C efflux capacity, which points out that HDL-C may be dysfunctional, which may contribute to residual cardiovascular disease risk. Even though HDL-C function is not yet a target in treatment of dyslipidemia, which limits application of this assay in everyday practice, we believe, that these novel findings of HDL-C function in Lithuanian population may contribute for future studies of new agents in dyslipidemia treatment.

It should be taken into account, that our results must be interpreted with some caution because this study has some potential limitations. First of all modest sample size led us to restriction of dividing population into subgroups and analyzing smaller variables and tendencies in between. Study population were found to be quite heterogeneous especially according age differences. In addition, the fact that some of participants were using statins may impact the results of our study. Further studies are needed.

## Conclusion

Majority of participants had below-average HDL-C efflux capacity. It was inversely associated with HDL-C concentration. Abnormal HDL function may be associated with residual cardiovascular risk in Lithuanian population.

## Data Availability

Data supporting the findings of this study are not publicly available, as the research is ongoing and further publications are being done.
